# Acute spinal subdural hematoma: A case report of spontaneous recovery from paraplegia

**DOI:** 10.1097/MD.0000000000020032

**Published:** 2020-05-08

**Authors:** Kazuya Yokota, Osamu Kawano, Hironari Kaneyama, Takeshi Maeda, Yasuharu Nakashima

**Affiliations:** a Department of Orthopaedic Surgery, Japan Labor Health and Welfare Organization Spinal Injuries Center; b Departments of Orthopaedic Surgery, Graduate School of Medical Sciences, Kyushu University, Japan.

**Keywords:** MRI, paraplegia, spinal cord injury, spinal subdural hematoma, spontaneous recovery

## Abstract

**Rationale:**

Spontaneous spinal subdural hematoma (SSDH) is a rare disease that can cause severe permanent neurological dysfunction. Here we present a case of spontaneous SSDH, in which a series of magnetic resonance images (MRIs) taken through the course of the disease facilitated understanding of the resolution process of the hematoma and the diagnosis of SSDH.

**Patient concerns:**

A 59-year-old male presented with sudden severe back pain and rapid onset of paraplegia. This symptom had continued developing while he was transferred to the emergency department. Initial physical examination showed flaccid paralysis of both lower limbs with areflexia and loss of all sensation below T6 bilaterally. MRI images showed an anterior subdural hematoma from C7 to T7 with spinal cord compression.

**Diagnosis:**

Based on MRI findings, the diagnosis was SSDH.

**Interventions:**

We chose conservative treatment of 1-week bed rest and intensive rehabilitation for the patient due to the presence of sacral sparing and the slight motor recovery at 24 hours after the onset.

**Outcomes:**

Frequent MRI images demonstrated that the spinal cord compression was surprisingly mitigated only 2 days and mostly absorbed 4 days after the onset. The patient's motor function was recovered completely and he was discharged after 8 weeks of hospitalization.

**Lessons:**

Our chronological MRI findings provide crucial information for diagnosing SSDH and also suggest that spinal surgeons should consider the potential option of a conservative approach for treating SSDH. Although prompt selection of a therapeutic strategy for SSDH could be challenging, the surgeons could observe the course of the patient's neurological status for a few days to detect signs of spontaneous recovery.

**Key Points**

- Acute spontaneous spinal subdural hematoma (SSDH) is a very rare condition.
- The treatment policy for SSDH is still controversial.
- Chronological MRI depicted a spontaneous decrease in the hematoma at only two days after the presentation of paraplegia.
- One-week of bed rest and intensive rehabilitation allowed the patient with SSDH to recover motor function completely from paraplegia.
- The presence of sacral sparing, the slight motor recovery after the onset, and the disappearance of back pain were key factors to determine conservative therapy for SSDH.

## 1 Introduction

Spinal subdural hematoma (SSDH) is a rare disease, and this pathological condition brings about devastating neurological dysfunction with significant morbidity and mortality.^[1]^ SSDH is mostly caused by trauma, lumbar puncture, and spinal surgery,^[2,3]^ whereas it can also occur spontaneously in patients with coagulating disorders underlying vascular malformation, neoplasms, and vulnerability of the vessel walls.^[4]^ Clinical symptoms of SSDH are characterized by the symptoms of spinal cord injury: motor, sensory, and/or autonomic dysfunction resulting from spinal cord compression.^[5]^

Previous reports have shown reviews of spontaneous SSDH,^[1,6,7]^ in which surgical decompression approaches, such as laminectomy and laminoplasty, are reportedly effective in achieving functional improvement for SSDH patients, whereas some SSDH patients showed no improvement after the application of surgical approaches.^[8,9]^ Additionally, previous reports have demonstrated complete recovery even after conservative treatment for SSDH patients.^[10]^ Although it is generally agreed that prompt evacuation should be performed before the permanent damage occurs in the spinal cord, the therapeutic strategy for SSDH still remains controversial.

Here, we report a case of SSDH presenting as sudden onset of back pain with paraplegia with no identifiable trauma. The patient's neurological dysfunction improved following conservative treatment. We successfully detected the resolution process of the subdural hematoma within only a week of the onset of presenting paraplegia, which could provide crucial insights to inform therapeutic policy and the diagnosis of SSDH.

## 2 Case report

A 59-year-old man presented with sudden severe back pain that started while he was smoking. The pain reached a peak in a few minutes, by which time the patient had become aware of insensitivity, numbness, and powerlessness in his legs. He called for an ambulance because of his persistent, severe paraplegia. This symptom had continued developing while he was transferred to the emergency department. He had no history of trauma, but he had a history of diabetes mellitus, hypertension, and coronary artery disease. He was taking prescribed antidiabetic drugs and oral antiplatelet agents, including aspirin (acetylsalicylic acid) and clopidogrel. The patient also had a history of smoking one pack of cigarettes per day for 40 years. Physical examination at the other emergency department showed flaccid paralysis of both lower limbs with areflexia and loss of all sensation below T6 bilaterally, excluding the perianal area. The patient's touch sensation was slightly retained at only the perianal area bilaterally; therefore, the initial neurological diagnosis was American Spinal Injury Association (ASIA) impairment scale (AIS) grade B.^[11]^ Eight hours after the onset, he was transferred to our specialized institute due to the previous findings about his paraplegia. He was still presenting flaccid paralysis of both legs at the time of transfer to our institute, whereas the back pain was already completely disappeared. Physical examination showed hypesthesia on the right side below T6 and the analgesia on the left side below T6, excluding perianal area. He had slight touch sensation, but not pinprick pain sensation, at the perianal area bilaterally. Both knee and ankle reflexes were absent, and Babinski sign was positive bilaterally. Rectal examination revealed a flaccid anal sphincter, and anal voluntary control was absent. The bulbocavernosus reflexes and anal wink response were both positive. He had no bowel or bladder control at the time of admission.

**Figure 1:**
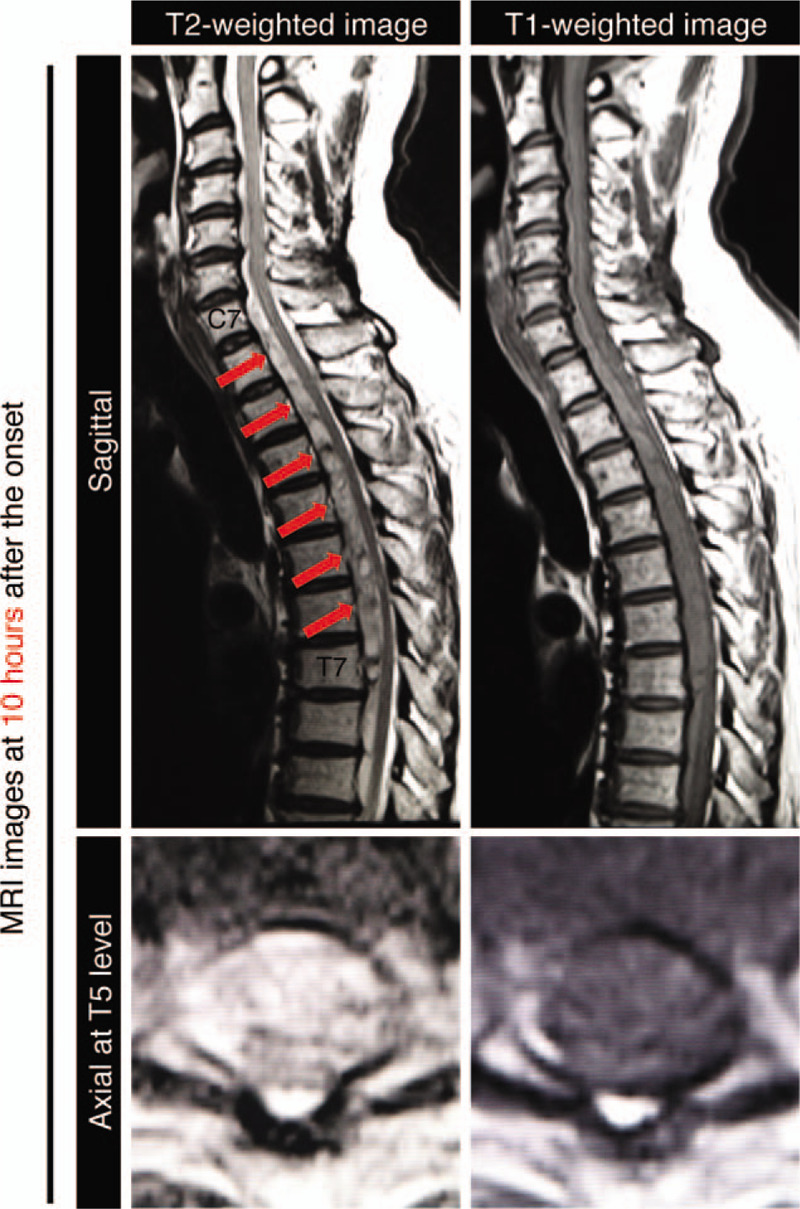
Magnetic resonance imagings (MRIs) of cervicothoracic spine at 10 hours after the onset of presenting paraplegia. T2-weighted and T1-weighted images revealed a long segment lesion of mixed isointensity and hyperintensity signals from C7 to T7. MRI images of the axial section at the T5 level showed the displacement of the spinal cord due to the lesion.

**Figure 2:**
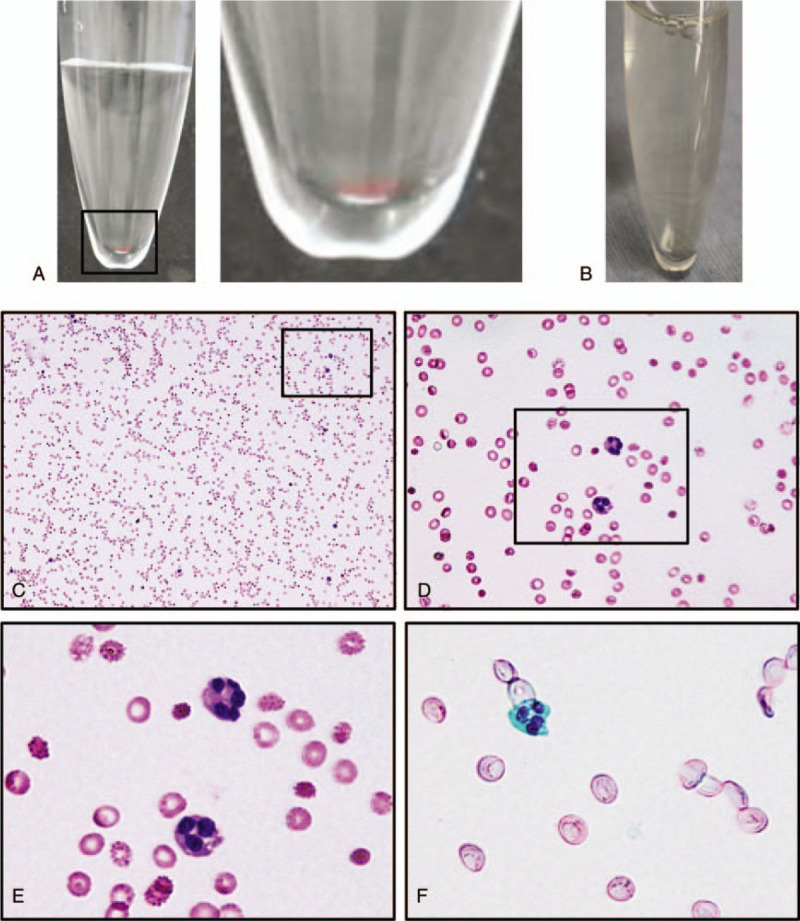
(A) Images of the collected cerebrospinal fluid (CSF) at 9 hours after the onset. The right image is a magnification of the boxed area. The CSF was slightly hemorrhagic. (B) Image of the collected CSF at 7 days after the onset, showing absence of hemorrhage and slightly yellow-tinged xanthochromia. (C) May-Giemsa stained sections of the CSF at 9 hours after the onset showed red blood cells and some multi-lobulated leukocytes. The images in (D) and (E) are magnifications of the boxed areas in (C) and (D), respectively. (E) Papanicolaou-stained sections of the CSF at 9 hours after the onset also showed the presence of multi-lobulated leukocytes.

The results of peripheral blood tests showed a white blood cells (WBC) count of 135 × 10^2^ cells/μL, a C-reactive protein level of 0.1 mg/dL, a platelet count of 27.5 × 10^4^ cells/μL, a prothrombin time of 13.8 seconds, an international normalized ratio (INR) of 1.15, and a partial thromboplastin time of 30.3 seconds. At 10 hours after the onset of the symptoms, a magnetic resonance imaging (MRI) scan of the cervicothoracic spine was performed (Fig. 1). In sagittal sections, an anterior subdural hematoma extending from C7 to T7 was detected. The hematoma was visualized in T1-weighted mixed isointense/hyperintense and T2-weighted mixed isointense/hyperintense images. The axial images displayed posterior displacement of the spinal cord due to the presence of the hematoma. To further corroborate that the location of hematoma is located in subdural space, the patient's cerebrospinal fluid (CSF) was collected, the appearance of which was slightly hemorrhagic (Fig. 2). May-Giemsa and Papanicolaou staining showed that his CSF contained red blood cells and multilobulated leukocytes. We also found that his CSF was already clear at 7 days after admission, which suggests that the bleeding in the subdural space was limited to the time of the onset and stopped quickly after the onset.

**Figure 3:**
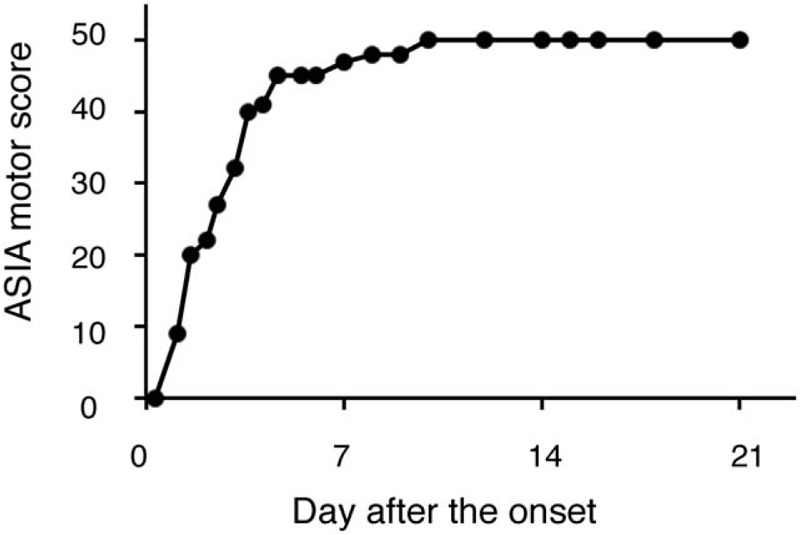
The time course of functional recovery according to the ASIA motor score in lower limbs. The patient achieved a perfect score at 10 days after the onset. ASIA = American Spinal Injury Association.

**Figure 4:**
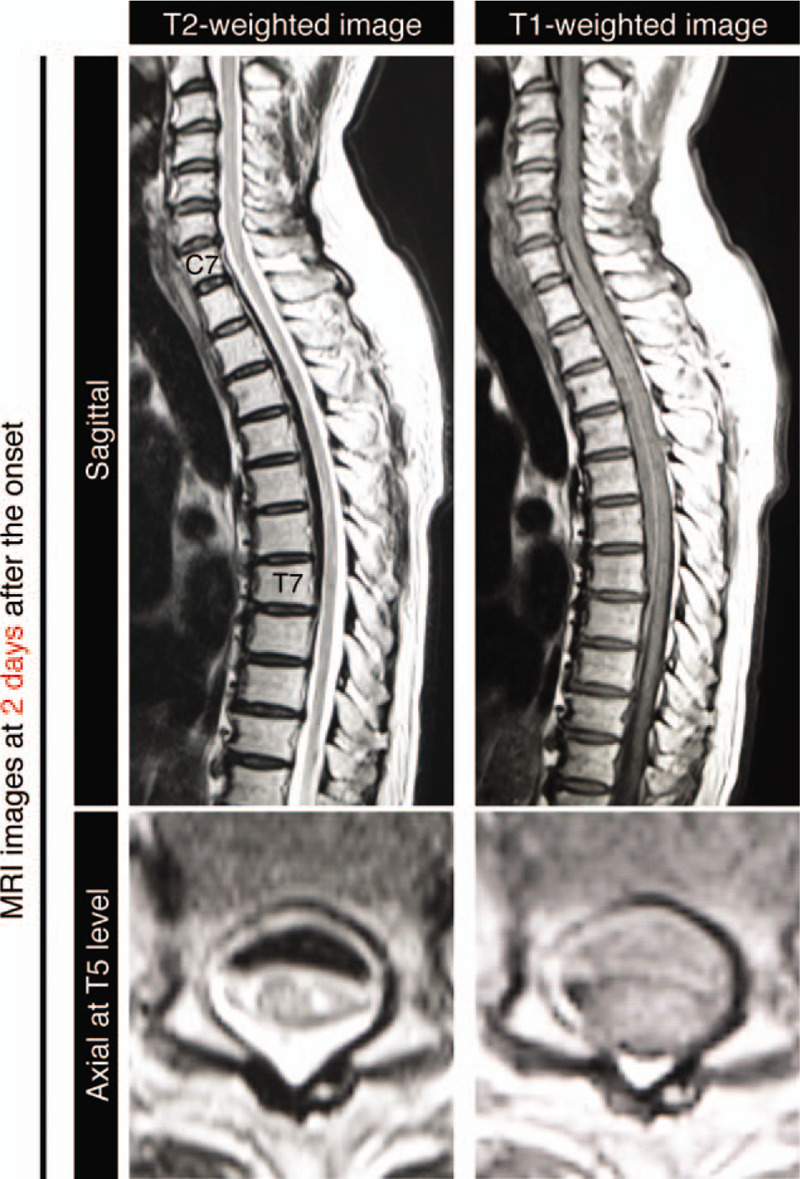
MRIs of the cervicothoracic spine at two days after the onset of presenting paraplegia. T2-weighted and T1-weighted images revealed that the compression of the spinal cord was released from the hematoma. The intensity of the lesion showed hypointensity in the T2-weighted images. MRI images of an axial section at the T5 level also showed the mitigated displacement of the spinal cord due to the hematoma.

**Figure 5:**
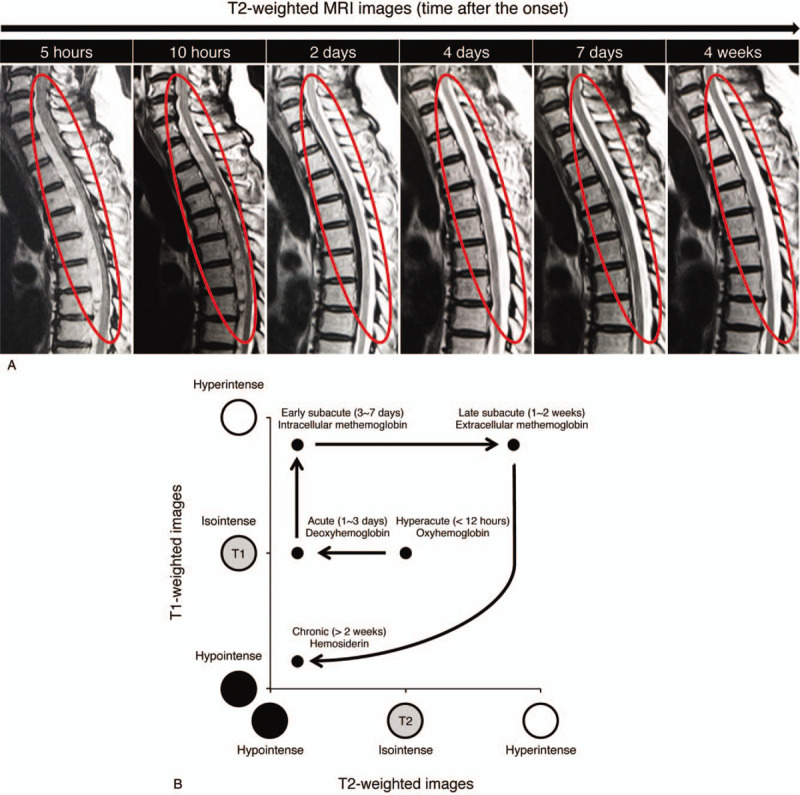
(A) Chronological MRI images demonstrated the process of the lesion resolution. At four days after the onset, the compression of the spinal cord was completely released from the lesion. There was no evidence of the recurrence of hematoma or intramedullary signal changes of the spinal cord at four weeks after the onset. (B) A schematic illustration shows magnetic resonance alternation of the hematoma at 5 stages according to our findings and those of others.

At 24 hours after the onset, his neurological score improved spontaneously from zero to nine on the ASIA motor scoring system (Fig. 3).^[11]^ We chose conservative management to treat the patient in our institute because his motor score had improved a little spontaneously and his sacral sparing was persistently observed from the initial admission. Our treatment policy included 1-week bed rest and an intensive rehabilitation program. He did not undergo any corticosteroid treatment or any surgical treatment. We had been paying attention to MRI signal changes, especially from the onset through 7 days after admission because the previous report showed the spontaneous resolution of the subdural hematoma at seven days after the onset.^[10]^ Remarkably, the hematoma of the patient had already decreased even at 2 days after the onset, which mitigated the compression of the thoracic spinal cord (Fig. 4). Furthermore, the hematoma was almost completely resolved at 4 days after the onset (Fig. 5A). The hematoma depicted in MRI images demonstrated the same signal patterns as those observed in the previous study showing signal change in patients with intraspinal hematoma in both T1-weighted and T2-weighted images (Fig. 5B).^[12]^ A lumbar tap revealed that his CSF was already clear and slightly yellowish at seven days after admission, which suggests that the bleeding in the subdural space was limited to the time of the onset and stopped quickly thereafter (Table 1). We further confirmed that the patient had no recurrence of hematoma at 4 weeks after the onset. In addition to this noticeable resolution process of the hematoma, we observed his gradual neurological recovery after post-onset admission. The neurological ASIA motor score showed complete recovery at 10 days after the onset (Fig. 3). From the viewpoint of the AIS, his neurological status improved to grade C at 1 day, to grade D at 3 days, and to grade E at 8 weeks after he first presented with paralysis (Table 2). Bladder management changed to intermittent self-catheterization at 9 days of admission, and urinary function was recovered completely at 7 weeks of admission. After 8 weeks of hospitalization, he was discharged from our institute and went home directly. He did not present any spasticity in the lower limbs or deterioration of neurological functions after recovery (Table 2).

**Table 1:**
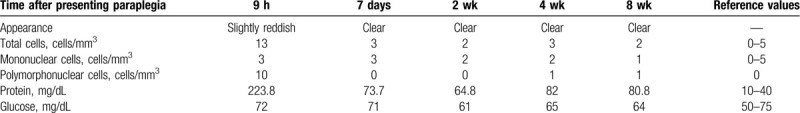
Cerebrospinal fluid status after the onset of spinal subdural hematoma.

**Table 2:**
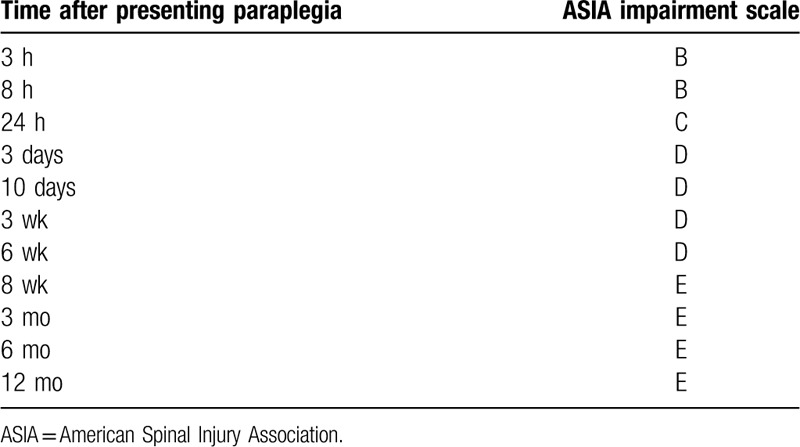
Neurological status after the onset of spinal subdural hematoma.

## 3 Discussion

This report presents sequential cervicothoracic MRI scans of a patient who had spontaneous SSDH with paraplegia and recovered completely following conservative treatment. The scans depicted the chronological signal alternation of the hematoma and demonstrated the resolution process of the hematoma, corresponding with the course of neurological improvement from an AIS grade B to an AIS grade E. We herein propose that conservative treatment for spontaneous SSDH is still a viable option with the potential to result in complete recovery even after presenting with paraplegia.

The uniqueness of the case in relation to the existing literature is that we successfully recorded the chronological resolution process of the subdural hematoma (Fig. 5). Interestingly, the hematoma compressing the patient's spinal cord had decreased in size only 2 days after the onset, when the compression of the spinal cord had already been released, as evidenced by the presence of CSF space posterior to the spinal cord (Fig. 4). To the best of our knowledge, this is the first report to describe evidence of spontaneous resolution of SSDH in such an acute phase.^[2,10]^ We also precisely described the functional improvement in parallel with the chronological MRI findings, providing the crucial insight that the surgeons could choose a therapeutic strategy based on the possibility of spontaneous resolution.

MRI is the standard modality in the evaluation of spinal cord injuries, visualizing not only the hematoma but also other spinal cord pathologies.^[13]^ Although MRI is able to depict the location and the chemical state of a hematoma,^[14,15]^ differential diagnosis between subdural hematoma and epidural hematoma is difficult, especially in the acute phase. We diagnosed this case as a subdural hematoma because epidural fat tissue without displacement of the dura was observed in the acute phase (Fig. 1). The hematoma would be considered to have occurred in the epidural space if dural displacement occurred toward the spinal cord, as discussed in previous literature.^[10]^ In addition to these characteristics, the shape of the epidural hematoma is biconvex, whereas the shape of the subdural hematoma is a crescent,^[16–18]^ which also corresponded with our findings, as displayed in Figure 4. However, the shape of the hematoma was not helpful as a diagnostic criterion 10 hours after the onset because the lesion actually seemed to be biconvex, as displayed in Figure 1. The developing range of the hematoma is also informative. SSDH generally spreads to several levels of the spine, whereas the epidural hematoma is limited to only a few levels.^[16]^ This case presented a wide range of levels, from C7 to T7, corroborating that the hematoma was located in the subdural space. The results of the collected CSF also supported the notion that the hematoma was located not in the epidural space but in the subdural space. We were not able to confirm a definite diagnosis of the patient as confidently as we could have if we had performed surgery and directly observed the intraspinal lesion, but the resolution process, the location of fat, the shape of the hematoma, and the range of the lesion in MRI images suggest that the hematoma had occurred in the subdural space.

The interpretation of blood in a lumbar puncture is not always straightforward. Interestingly, previous literature demonstrates how to distinguish traumatic lumbar puncture from true subdural/subarachnoid hemorrhage.^[19,20]^ In cases in which consecutive CSF samples gradually become clearer a traumatic tap is likely, whereas persistent discoloration suggests true intradural hemorrhage. Yellow discoloration of the CSF indicates xanthochromia, which persists from 1 to 2 weeks after intradural hemorrhage due to increased bilirubin levels. Although useful, it is still difficult to infer the location of a hematoma based on CSF findings alone. In the present case, the appearance of the CSF in Figure 2A does not suggest a traumatic tap which would be readily visible by the strikingly reddish discoloration.^[20]^ In addition, we collected the CSF from the lumbar level in which level the hematoma was not present, excluding the possibility of an epidural hematoma. The absence of a gradual clearing of blood in serially collected CSF at 9 hours after the onset indicates that the puncture was not traumatic. Furthermore, the mild elevation of CSF protein levels at 7 days suggests the existence of bilirubin, which is transformed from hemoglobin in the subdural space.^[21]^ In fact, the appearance of CSF at 7 days exhibited the slight yellow tinge of xanthochromia, indicating that the hemorrhage was located in the subdural space. Although the interpretation of blood in the CSF collected through lumbar puncture studies is not always straightforward, we believe that it was helpful in this case to diagnose the location of the hemorrhage in the subdural space.

The patient underwent fairly rapid improvement within 24 hours although he presented complete and flaccid paralysis immediately after the onset. MRI scans showed that the spinal cord was highly compressed at 10 hours after the onset and the compression was released at 2 days even if the hematoma still existed, suggesting that the compression of the spinal cord could have been already mitigated at 24 hours.^[22]^ Determining a treatment policy for SSDH is challenging because SSDH is a rare disease. It is not feasible to collect enough data from SSDH patients to perform a prospective and randomized study. Previous reports have suggested that the effectiveness of surgical treatment is uncertain because some SSDH patients present with persistent paraplegia even after the decompression of the lesion.^[3,6]^ These reports included a group of patients with complete paralysis, whereas our patient presented with incomplete paralysis. Our findings and those reports suggest that the SSDH patients with incomplete paraplegia may not necessarily require surgery. Importantly, especially in cases with wide range of hematoma, an extensive decompression in the affected spine is crucial to release the compression of the spinal cord, which is rather invasive and possibly causes unexpected complications. Given that the recovery rate for SSDH following surgical treatment is not higher than that achieved with conservative therapy, even if the operation was performed within 48 hours,^[7]^ the surgeons on standby could stay alert to the potential of better spontaneous progress in physical findings, at least for a few days after the onset.

One of the limitations of this report is that the etiology of SSDH remains unknown. The underlying cause of SSDH in this case is difficult to pinpoint because the patient had several factors that made him susceptible to hemorrhagic events. He was taking antiplatelet drugs for 5 years and was also being treated for diabetes mellitus, which makes small vessels more vulnerable to minor environmental change and susceptible to vascular complications.^[23]^ In addition, the patients had a long history of smoking. The symptoms actually presented while he was smoking, suggesting that smoking may have precipitated dysfunction of the spinal vessels and possibly led to their rupture.^[24]^ Considering that patients’ complicated history, we believe that multiple factors in this patient's background contributed to the onset of the condition.^[7]^ We also must point out that neither angiography nor delayed MRI were performed to exclude vascular malformation that may have precipitated SSDH. Our facility is limited in equipment and radiological staff, limiting our imaging to T1 and T2-weighted MRI and plain CT scans. As far as we could surmise based on the images from the MRI and CT scans, no apparent neoplastic lesions were observed in the levels in which the hematoma was observed.

In conclusion, we report a case of SSDH whose diagnostic features on MRI scans were the location, the presence of the intraspinal fat, the shape of the hematoma, and the range of spinal levels affected. The alternation of signal intensity in MRI images was consistent with those found in previous studies. The disappearance of the back pain possibly indicated that there was no more extension of the hematoma. Motor recovery, even if slight, could be a predictor of successful conservative therapy. Our chronological observations suggest that functional improvement after the onset of SSDH is associated with resolution of the hematoma and mitigated compression of the spinal cord. In the presented case, the resolution of the hematoma could be found even in the acute phase, within 4 days after the onset; therefore, spine surgeons could possibly consider the conservative options for dealing with paraplegia caused by SSDH.

## Acknowledgments

The authors thank the patient featured in this case report for generously allowing the publication of personal medical information.

## Author contributions

**Conceptualization:** Kazuya Yokota, Osamu Kawano, Hironari Kaneyama, Takeshi Maeda, Yasuharu Nakashima.

**Data curation:** Kazuya Yokota, Osamu Kawano, Hironari Kaneyama, Takeshi Maeda, Yasuharu Nakashima.

**Formal analysis:** Kazuya Yokota, Osamu Kawano, Hironari Kaneyama, Takeshi Maeda, Yasuharu Nakashima.

**Investigation:** Kazuya Yokota, Osamu Kawano, Hironari Kaneyama, Takeshi Maeda, Yasuharu Nakashima.

**Supervision:** Kazuya Yokota, Osamu Kawano, Hironari Kaneyama, Takeshi Maeda, Yasuharu Nakashima.

**Validation:** Kazuya Yokota, Osamu Kawano, Hironari Kaneyama, Takeshi Maeda, Yasuharu Nakashima.

**Writing – original draft:** Kazuya Yokota.

**Writing – review & editing:** Kazuya Yokota, Osamu Kawano, Hironari Kaneyama, Takeshi Maeda, Yasuharu Nakashima.
